# Sarcoplasmic reticulum Ca^2+^‐induced Ca^2+^ release regulates class IIa HDAC localization in mouse embryonic cardiomyocytes

**DOI:** 10.14814/phy2.13522

**Published:** 2018-01-22

**Authors:** Sari Karppinen, Sandra L. Hänninen, Risto Rapila, Pasi Tavi

**Affiliations:** ^1^ Department of Biotechnology and Molecular Medicine A.I. Virtanen Institute for Molecular Sciences University of Eastern Finland Kuopio Finland; ^2^ Institute of Biomedicine Department of Physiology and Biocenter Oulu University of Oulu Finland

**Keywords:** Calcium, cardiomyocyte differentiation, gene expression, histone deacetylase, sarcoplasmic reticulum

## Abstract

In embryonic cardiomyocytes, sarcoplasmic reticulum (SR)‐derived Ca^2+^ release is required to induce Ca^2+^ oscillations for contraction and to control cardiac development through Ca^2+^‐activated pathways. Here, our aim was to study how SR Ca^2+^ release regulates cytosolic and nuclear Ca^2+^ distribution and the subsequent effects on the Ca^2+^‐dependent localization of class IIa histone deacetylases (HDAC) and cardiac‐specific gene expression in embryonic cardiomyocytes. Confocal microscopy was used to study changes in Ca^2+^‐distribution and localization of immunolabeled HDAC4 and HDAC5 upon changes in SR Ca^2+^ release in mouse embryonic cardiomyocytes. Dynamics of translocation were also observed with a confocal microscope, using HDAC5‐green fluorescent protein transfected myocytes. Expression of class IIa HDACs in differentiating myocytes and changes in cardiac‐specific gene expression were studied using real‐time quantitative PCR. Inhibition of SR Ca^2+^ release caused a significant decrease in intranuclear Ca^2+^ concentration, a rapid nuclear import of HDAC5 and subnuclear redistribution of HDAC4. Endogenous localization of HDAC5 and HDAC4 was mostly cytosolic and at the nuclear periphery, respectively. Downregulated expression of cardiac‐specific genes was also observed upon SR Ca^2+^ release inhibition. Electrical stimulation of sarcolemmal Ca^2+^ influx was not sufficient to rescue either the HDAC localization or the gene expression changes. SR Ca^2+^ release controls subcellular Ca^2+^ distribution and regulates localization of HDAC4 and HDAC5 in embryonic cardiomyocytes. Changes in SR Ca^2+^ release also caused changes in expression of the developmental phase‐specific genes, which may be due to the changes in HDAC‐localization.

## Introduction

Differentiating cardiomyocytes lack the specialized structures normally found in terminally differentiated cardiomyocytes, which enable functional coupling between the sarcolemmal (SL) Ca^2+^‐influx and sarcoplasmic reticulum (SR) Ca^2+^‐release (Buckingham et al. 2005). Many of these structures are required for the strict spatiotemporal synchronization of subcellular Ca^2+^ signals, hence the immature cardiomyocytes must rely on Ca^2+^ signals with a high degree of variance in both the spatial and temporal domains (Korhonen et al. 2010; Louch et al. 2015).

Due to the perinuclear dominance of triggering Ca^2+^ oscillations in the embryonic cardiomyocytes (Korhonen et al. 2010; Rapila et al. 2008) the role of SR Ca^2+^ release in the regulation of nuclear Ca^2+^ signaling is of interest. Previous studies have found that in addition to the cytosolic Ca^2+^ fluxes passively diffusing into the nucleus, the nucleus also contains its own sources of Ca^2+^, such as the nuclear envelope. In addition, the perinuclear SR also contributes to the changes in nuclear Ca^2+^ levels (Ibarra et al. 2014). Furthermore, the ryanodine receptor type 2 (RyR2)‐ and inositol 1,3,4‐trisphosphate receptor (IP_3_R)‐containing nucleoplasmic reticulum has been shown to be present in both neonatal and adult cardiomyocytes, suggesting that the nucleus might be able to control, or at least regulate, its Ca^2+^ levels (Guatimosim et al. 2008).

In neonatal cardiomyocytes, two ways were found to elicit Ca^2+^ transients, each of them having very different effects on nuclear Ca^2+^ signaling (Nakao et al. 2015). When the cells were electrically stimulated, the nuclear Ca^2+^ oscillations resembled the cytosolic Ca^2+^ signals, and they were RyR‐initiated mostly from the cytosol. Receptor stimulation‐induced Ca^2+^ release on the other hand caused IP_3_R‐mediated Ca^2+^ release, which had a much larger effect on nuclear than cytosolic Ca^2+^ transients (Nakao et al. 2015). Hence, the mode by which Ca^2+^ is mobilized has a significant effect on how the nuclear Ca^2+^ level is modulated. This difference is quite important when studying Ca^2+^ signaling in embryonic cardiomyocytes, where the normal physiological Ca^2+^ oscillations are initiated by continuous ET‐1/IP_3_‐mediated stimulation of SR Ca^2+^ release rather than electrical stimulation (Karppinen et al. 2014). Hence, any change in Ca^2+^ signaling in embryonic cardiomyocytes could have relatively large effects also on the nuclear Ca^2+^ oscillations.

Various cardiac malformations and embryo lethality before mid‐gestation have been observed with RyR, SR Ca^2+^ ATPase (SERCA), IP_3_R and calreticulin (Calr) knock‐out mouse models (Takeshima et al. 1998; Mesaeli et al. 1999; Periasamy et al. 1999; Andersson et al. 2009; Uchida et al. 2010), suggesting a link between intracellular Ca^2+^ release, cardiac‐specific gene expression and cardiac development. Many of the signaling pathways essential for cardiac development are indeed regulated by Ca^2+^. For example, class IIa histone deacetylaces (HDACs) are essential epigenetic factors that function by deacetylating histone tails and thereby condensing the chromatin structure leading to repression of their target genes (Kuo and Allis 1998). During cardiac differentiation and development, their role is especially important. H3K27 acetylation (with H3K4me1) within the gene enhancer regions is required for enhancer activation, which in turn correlates with cardiac‐specific programmes, especially at the cardiac progenitor‐stage or in cardiomyocytes (Wamstad et al. 2012). Hence, HDACs can regulate the open/closed status of the chromatin, thereby regulating the availability of those regions to the transcription factors and the transcriptional machinery.

The repressive action of the class IIa HDACs, especially HDAC4 and HDAC5, is dependent on their nuclear localization, which in turn depends on the relative strengths of nuclear Ca^2+^ signals. Increased Ca^2+^ concentration stimulates Ca^2+^‐activated kinases such as CaMK or PKD (Vega et al. 2004; McKinsey et al. 2000; McKinsey 2007; Monovich et al. 2010), which phosphorylate class IIa HDACs. This enables their nuclear export (Backs and Olson 2006; Bossuyt et al. 2008) and subsequently de‐represses their target genes. Class IIa HDACs have been shown to regulate expression of cardiac‐specific genes, such as transcription factors (TF) *MEF2C, GATA4* and *Nkx2.5* that are essential in cardiomyocyte differentiation (Karamboulas et al. 2006; Kee and Kook 2011). They also function in cooperation with the same TFs to regulate expression of various other cardiac‐specific genes (Backs and Olson 2006).

This study aimed to determine if SR Ca^2+^ release can regulate the intranuclear Ca^2+^ concentration ([Ca^2+^]_nuc_) in mouse embryonic cardiomyocytes. Secondly, we aimed to study how changes in Ca^2+^ distribution affect the Ca^2+^‐dependent nuclear signaling that can lead to changes in class IIa HDAC localization and cardiac‐specific gene expression in embryonic cardiomyocytes.

## Materials and Methods

### Cell Isolation and Culturing

The method for cell isolation and culturing of the E9‐11 embryonic cardiomyocytes has been described previously (Rapila et al. 2008). Shortly, pregnant females were sacrificed for embryo collection between E9‐11. The dissected ventricles were incubated in pancreatin/collagenase‐solution to dissociate the cells. The cells were plated on laminin‐coated glass‐bottom petri dishes and cultured for 18–24 h. Due to the small amount of cells/ventricle, the cultures were not confluent. However, all the measurements were conducted on areas where the cells were connected. No single cells were measured. Two mouse strains, CD‐1 from the Centre for Experimental Animals at the University of Oulu and C57BL/6JOlaHsd from the National Laboratory Animal Centre at the University of Eastern Finland were used in this study. CD‐1 strain was used in all the experiments, with some additional Ca^2+^ mobilization and immunolabeling experiments performed at the University of Eastern Finland, using the C57BL/6JOlaHsd strain. In these experiments, where two different mouse strains were used, the results were comparable between the mouse lines. All experimental designs were approved by the Animal Use and Care Committee of the University of Oulu and the National Animal Experiment Board in Finland.

### Confocal Ca^2+^ imaging

Ca^2+^ imaging with simultaneous electrical stimulation was performed as described in a previous study (Rapila et al. 2008). To study changes in subcellular Ca^2+^ mobilization, Fluo‐4‐labeled, spontaneously active cells were imaged on a confocal microscope (with a 60x objective lens) on an imaging plane where all regions of interest (sarcolemma, perinuclear SR and nucleus) were clearly visible. The cells were perfused with culture medium containing a pharmacological inhibitor and simultaneously stimulated electrically (1 msec pulses, frequency 0.5 Hz, to stimulate Ca^2+^ influx through the cell membrane) and line‐scanned periodically to observe changes in subcellular Ca^2+^ signals. Pharmacological interventions used were thapsigargin (10 *μ*mol/L, Ascent Scientific, Avonmouth, UK), ryanodine (50 *μ*mol/L, Ascent Scientific, Avonmouth, UK) and Ca^2+^ ionophore, A23187 (1 *μ*mol/L, Sigma‐Aldrich, St. Louis, MO). As we were interested on the acute effect of these pharmacological interventions, the cells were imaged with the treatment until Ca^2+^ oscillations stopped or changes in Ca^2+^ distribution could be clearly observed (≤20 min). The line scan images were analyzed with the FluoView 1000 (Olympus, Tokyo, Japan) and Origin 2016 (OriginLab Co., Northampton, MA) programs. Fluo‐4 fluorescence intensity is expressed as an F/F_0_ ‐ratio, where F is the background subtracted fluorescence intensity and F_0_ is background subtracted minimum fluorescence value measured from each region at rest at control conditions. Fluorescent intensity in the three different intracellular locations was normalized to its own F_0_ value. Changes in Ca^2+^ signals were compared within each subcellular localization (control vs. treatment). No comparison between the localizations was conducted as this would have required calibration of the fluorescent dye at each subcellular localization.

### Immunofluorescence Labeling and Microscopy

E9‐11 cardiomyocytes were grown for 24–48 h on glass‐bottom Petri dishes to obtain spontaneously contracting cultures. The cells were then incubated for 30 min at 37°C with 0.5 Hz electrical pacing in a C‐PACE Cell Culture Stimulator (IonOptix Ltd, Dublin, Ireland) with or without pharmacological intervention (1 *μ*mol/L A23187, 50 *μ*mol/L ryanodine or 10 *μ*mol/L thapsigargin) and then immediately fixed and labeled, using a method described previously (Karppinen et al. 2016). Primary antibodies used were rabbit anti‐HDAC4 (Imgenex, Novus Biologicals, Littleton, CO, Cat # IMG‐338, 1:250 dilution) and rabbit anti‐HDAC5 (Sigma Aldrich, St Louis, MO, Cat # H9663, 1:250 dilution) with Alexa Fluor 488, chicken anti‐rabbit (Invitrogen, Waltham, MA, 1:750 dilution) secondary labeling. Images of the cells were taken immediately after staining with a confocal microscope (excitation 488 nm, emission 520–620 nm with 40x and 60x objective lenses; FV1000; Olympus, Tokyo, Japan) at 0.198 or 0.132 *μ*m/pixel spatial resolutions.

The images were analyzed using two different approaches. HDAC5 localization is expressed as a nucleus to cytosol‐ratio (n/c‐ratio). Here, after background subtraction, fluorescence measured in representative cytosolic and nuclear regions was used to calculate the n/c ratio. HDAC4 on the other hand was mainly nuclear, therefore the fluorescent signal was measured from the nuclear periphery and the center of the nucleus. These values were then normalized to the background subtracted cytosolic fluorescence.

### Plasmid transfection and protein translocation imaging

After culturing for 18–24 h, E9‐11 cardiomyocytes were transfected with a plasmid containing HDAC5‐GFP (a generous gift from Dr. Rhonda Bassel‐Duby, UT Southwestern Medical Center at Dallas), using Lipofectamine 2000 (Invitrogen) according to the manufacturer's instructions. Briefly, cells cultured on glass bottom culturing dishes were transfected with 100 *μ*L Opti‐MEM (Invitrogen) containing 0.2 *μ*g HDAC5‐GFP‐plasmid and 2 *μ*L Lipofectamine 2000 (Invitrogen). After a 5‐h incubation at 37°C, the medium was changed to Dulbecco's Modified Eagle Medium (DMEM) containing 10% fetal bovine serum (FBS, Sigma Aldrich) and 1% penicillin‐streptomycin (PS, Invitrogen) and the cells were cultured for additional 18–24 h to allow expression of the chimeric protein.

A confocal inverted microscope (FluoView 1000; Olympus, Tokyo, Japan) was used to take images of the HDAC5‐GFP with excitation at 488 nm and emission collected at 500–600 nm through a 60x objective lens. A steady temperature of 34°C was maintained by continuous superfusion with preheated culturing medium (without FBS and PS, pH 7.4, bubbled with 95% O_2_/5% CO_2_). Electrical stimulation (0.5 Hz) was maintained throughout the experiments with two platinum electrodes placed on opposing sides of the plate. The change in cytosolic versus nuclear fluorescence with pharmacological interventions (50 *μ*mol/L ryanodine or 10 *μ*mol/L thapsigargin) was followed over time and results expressed similarly to the immunolabeled HDAC5, as a n/c – ratio.

### RNA extraction and RT‐qPCR

Tissue samples (ventricles) from mouse embryos at different developmental stages (E8.5–neonatal) were used in these experiments. RNA extraction, cDNA synthesis and RT‐qPCR were performed as previously described (Karppinen et al. 2016). The *C*_t_ values from each reaction were first standardized to their own standard plot and then normalized to the housekeeping gene, 18S rRNA, quantified from the same samples (expression of class IIa HDACs at different developmental stages). When comparing the effect of SR Ca^2+^ release inhibition to cardiac‐specific gene expression, the results were further normalized to the control in each group. The primers and fluorescent probes are listed in Table 1.

**Table 1:** Primers and fluorogenic probes

Name	Sense primer (forward)	Antisense primer (reverse)	Fluorogenic probe
*Calr*	ctgcttggcctcctcgg	ccaagaactgctctttgaaatagatg	tggccgccgcagaccctg
*Casq2*	ggaacatcaaagacccaccct	tcgtcttcccatgtttcaaaca	cgtcgcttgcgcccagagg
*GATA4*	ggctatgcatctcctgtcactca	ggaccaggctgttccaagag	catcacaggccagctccaagcagg
*HDAC4*	cacactcctctacggcacaaatc	aggaagcctgacgaacactga	tctcaacagacagaaactggacagctcgc
*HDAC5*	gtctccactgggtggctattct	gtcatgagctgcctggtcaa	tcaccgccagatgttttggcca
*HDAC7*	tgtcagacccaagtcctcaaca	tgtttcagcatcaccgagtca	ctcagagacacctgctacagggctggtc
*HDAC9*	caaccaatggaaagaatcattcc	tgggcagccgtgtacca	tgggccgccatcccaagc
*MEF2c*	ggaacacgcctgtcacctaac	atgagtgccatacgccaatg	agcacgctcacaaacctgcaggc
*Nkx2.5*	ccctgtccctcagatttcaca	aaagtgggatggatcggagaa	cctcgcgcaggcctgggac
*18S rRNA*	tggttgcaaagctgaaacttaaag	agtcaaattaagccgcaggc	cctggtggtgcccttccgtca

### Statistics

The statistical significance of differences was analyzed, using the paired or two‐sample *t*‐test or one‐way analysis of variance with Bonferroni's post hoc test and Levene's test for equal variance. Results are expressed as mean ± SEM.

## Results

### Functional SR Ca^2+^ release is necessary for nuclear Ca^2+^ signaling

**Figure 1:**
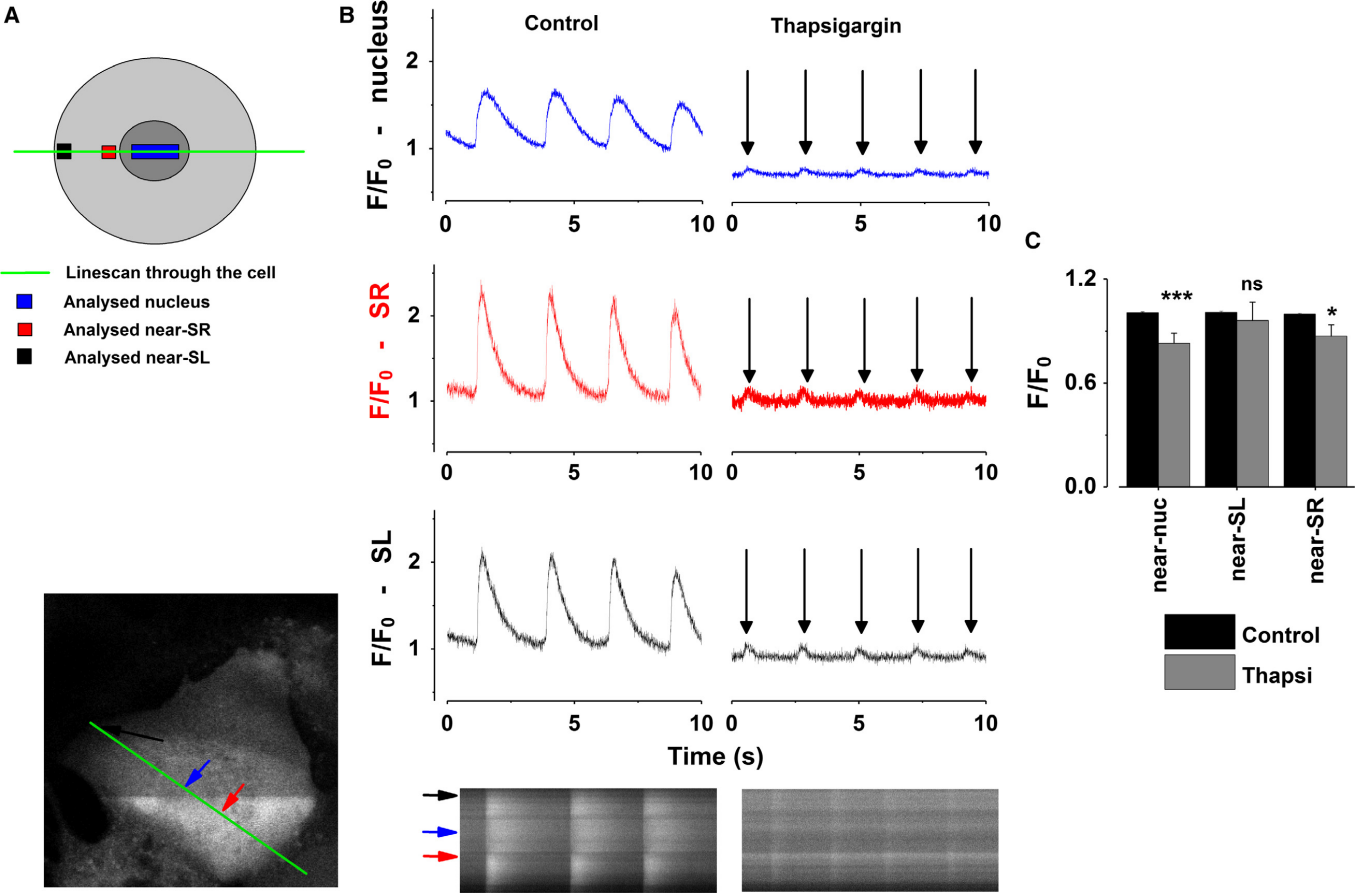
Sarcoplasmic reticulum Ca^2+^ release is required for nuclear Ca^2+^ mobilization. (A) A schematic diagram of a cell showing the locations within the line‐scan where the regions of interest were measured. The green line depicts the line‐scan, blue the region within that line accounts for the nuclear region, and the black and red regions near‐ sarcolemmal and near‐SR, respectively. (B) Representative Ca^2+^ signals in control conditions (spontaneously active) at different intracellular localizations (blue: nucleus, red: near‐SR, black: near‐SL Ca^2+^ influx) and with simultaneous depletion of the SR and electrical stimulation. The arrows denote the 0.5 Hz electrical stimuli. (C) Mean baseline Fluo4‐fluorescence (F/F_0_) in control conditions and in cells where SR Ca^2+^ has been depleted, using thapsigargin (10 *μ*mol/L, treated until the spontaneous activity ceased, up to 20 min). The values were normalized to the fluorescence intensity of each localization at control conditions. All results are expressed as a mean ± SEM *P < 0.05 (*n* = 12). SR, sarcoplasmic reticulum; SL, sarcolemmal.

In spontaneously active embryonic cardiomyocytes strong Ca^2+^ oscillations are observed throughout the cell. Depletion of the SR Ca^2+^ stores using thapsigargin (10 *μ*mol/L) caused the SR‐derived Ca^2+^ oscillations to stop, similarly as has been shown by previous studies (Rapila et al. 2008; Korhonen et al. 2008; Sasse et al. 2007; Viatchenko‐Karpinski et al. 1999). This occurred despite the electrical stimulation (Fig. 1B, right panel), which is normally able to stimulate Ca^2+^‐induced Ca^2+^ release (CICR) from the SR. Here, stimulation was possible for a short amount of time and Ca^2+^ oscillations stopped as soon as the SR was depleted.

Depletion of the SR decreased diastolic Ca^2+^ levels both in the cytosolic region near the SR and within the nucleus (17.4% and 12.7%, respectively, relative to their own resting Ca^2+^ levels, Fig. 1C). Hence, perinuclear SR Ca^2+^ release not only is the main source of the cytosolic Ca^2+^ oscillations, but it can also strongly regulate [Ca^2+^]_nuc_, with the changes in nuclear Ca^2+^ levels mirroring changes in SR Ca^2+^ mobilization.

### Developmental expression and localization of class IIa HDACs

**Figure 2:**
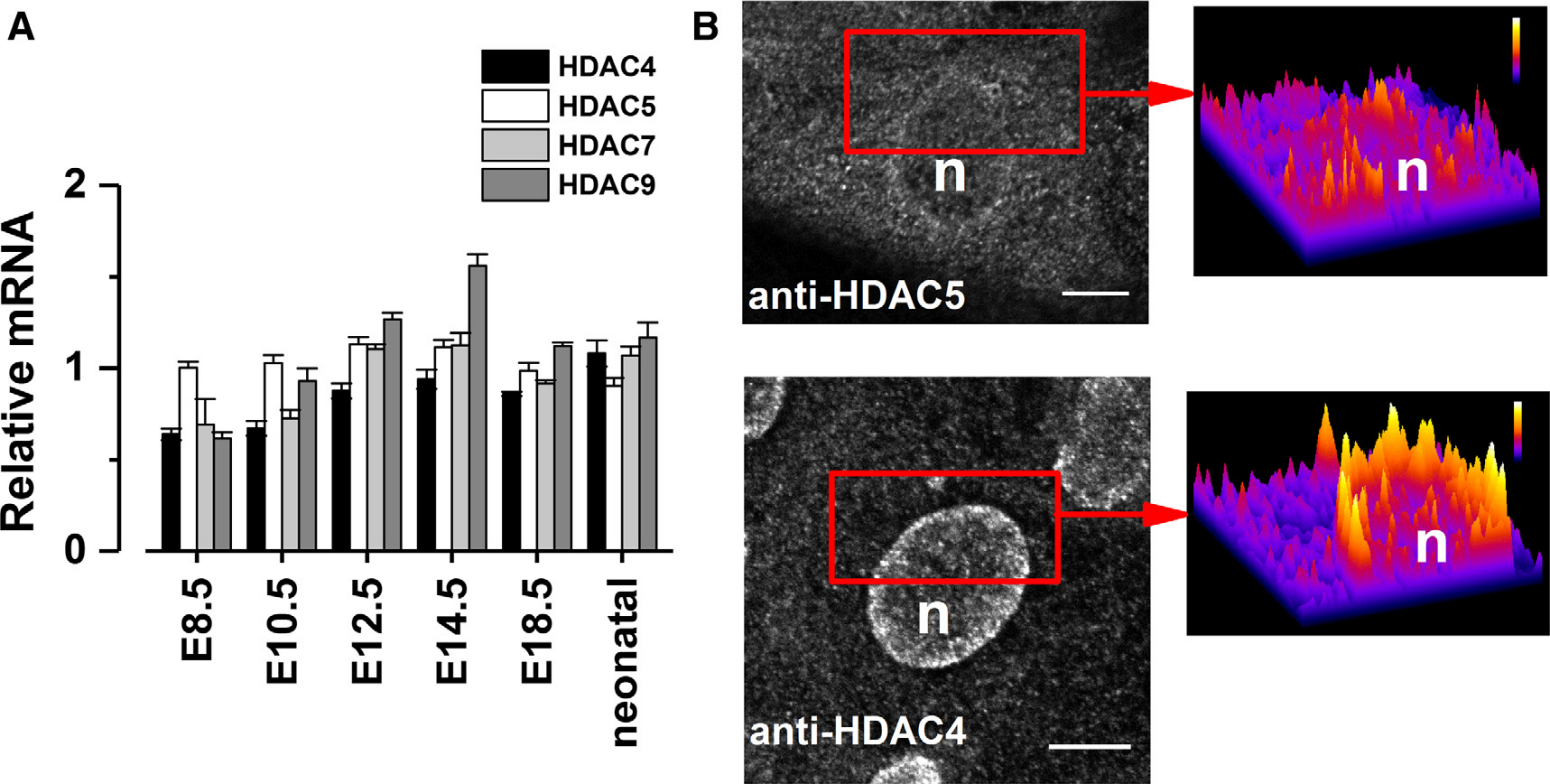
Expression and subcellular distribution of HDACs in the embryonic heart. (A) RT‐qPCR was used to study relative expression of class IIa HDAC mRNA in the developing mouse hearts at six different developmental stages (from E8.5 to neonatal mice, *n* = 6 in each group). (B) The black‐and‐white microscope images show the endogenous subcellular distribution of HDAC4 and HDAC5 in spontaneously active E9‐11 cardiomyocytes (*n* = nucleus, scale bar 10 *μ*m). The false‐color 3D‐image on the right shows a section of the microscope image to better show the cytosolic and nuclear distribution of these proteins. HDACs: histone deacetylases.

Class IIa HDACs are some of the best‐known Ca^2+^‐activated regulators of cardiac‐specific gene expression (Wu et al. 2006; Arantes et al. 2012) hence their presence in the developing heart was of interest. Here, expression of all class IIa HDAC isoforms was studied at different developmental stages, from the early embryonic to the neonatal phase (Fig. 2A). These data show that all the class IIa isoforms are present in cardiac tissue at every developmental stage studied.

Among these HDACs, previous studies have shown that isoforms 4 and 5 specifically regulate the developmental cardiac gene pattern upon nuclear [Ca^2+^] changes (Wu et al. 2006; Ito et al. 1991; Frey and Olson 2003; Zima et al. 2007) hence these two were chosen to be studied further. Interestingly, immunofluorescent labeling of these proteins in electrically stimulated E9‐11 cardiomyocytes shows HDAC4 to be localized mainly in the nuclear periphery, whereas HDAC5 is evenly distributed throughout the cells (Fig. 2B).

### Localization of the endogenous HDACs is dependent on SR Ca^2+^ release

**Figure 3:**
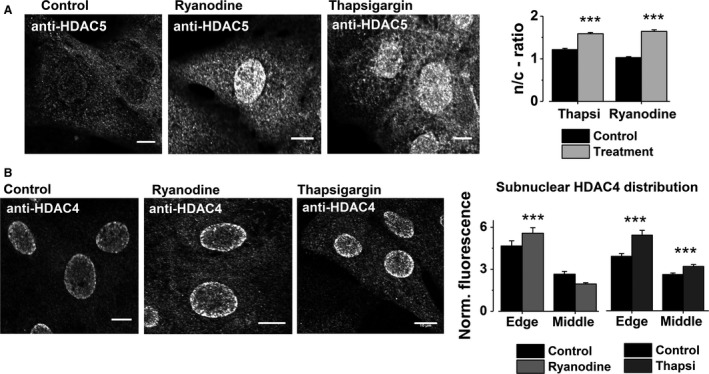
The effect of SR Ca^2+^ release inhibition on subcellular distribution of HDAC4 and HDAC5. (A) Representative immunofluorescent images of HDAC5 in embryonic cardiomyocytes in control conditions (control for ryanodine, *n* = 324 and for thapsigargin, *n* = 198) and with either SR Ca^2+^ release inhibition (ryanodine, 50 *μ*mol/L, *n* = 398) or SR depletion (thapsigargin, 10 *μ*mol/L, *n* = 239). The results are expressed as a mean nucleus/cytosol (n/c)‐ratio of the HDAC5 fluorescent intensity (±SEM, scale bar 10 *μ*mol/L). (B) Representative images of HDAC4‐stained embryonic cardiomyocytes in control conditions (*n* = 64 and *n* = 108, ryanodine and thapsigargin controls, respectively) and with either ryanodine (50 *μ*mol/L, *n* = 63) or thapsigargin treatment (10 *μ*mol/L, *n* = 83) (scale bar 10 *μ*m). In the HDAC4 experiments, nuclear fluorescence was first normalized to the cytosolic signal and fluorescence intensity was then measured in two locations within the nucleus; close to the nuclear membrane (edge) and the middle of the nucleus. The results are expressed as mean ± SEM. All the groups (both in A and B) were electrically stimulated throughout the experiments (0.5 Hz, 30 min). ***P < 0.001. HDACs: histone deacetylases; SR, sarcoplasmic reticulum; SL, sarcolemmal.

In paced E9‐11 cardiomyocytes, inhibition of SR Ca^2+^ release with either ryanodine (50 *μ*mol/L) or thapsigargin (10 *μ*mol/L), resulted in strong HDAC5 nuclear import and subnuclear redistribution of HDAC4 toward the nuclear periphery (Fig. 3A and B).

**Figure 4:**
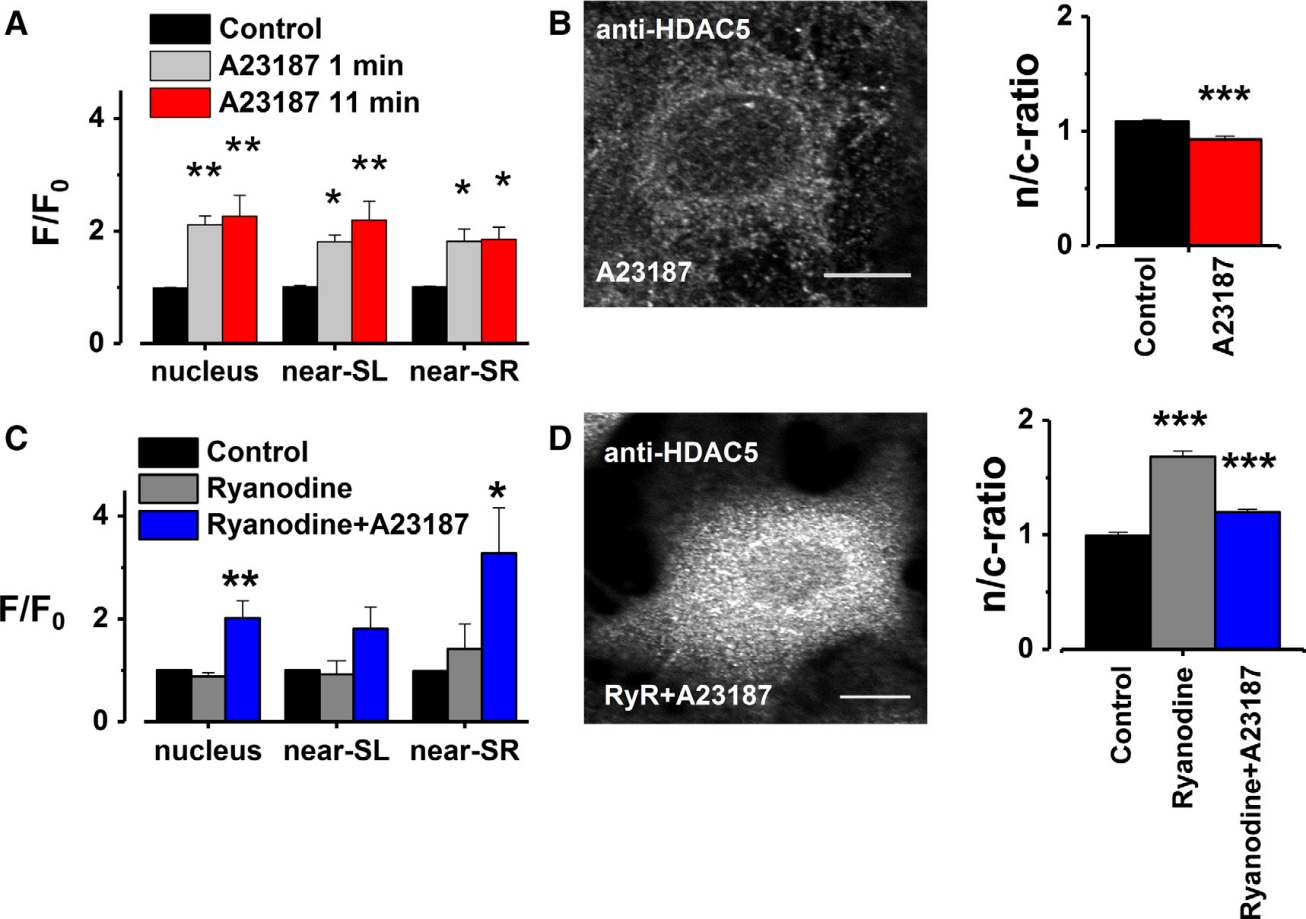
Strong Ca^2+^ influx through SL can influence both [Ca^2+^]_nuc_ and HDAC5 localization. An increase in membrane permeability to Ca^2+^ had a strong effect on (A) baseline Ca^2+^ levels at different intracellular localizations (*n* = 6). It also caused (B) HDAC5 nuclear export (control, *n* = 74; 1 *μ*mol/L A23187, *n* = 111). The representative image shows an A23187‐treated, HDAC5‐labeled myocyte. Similar effects were also observed at (C) the baseline Ca^2+^ level (*n* = 6) and (D) HDAC5 localization, when the cells were treated with either ryanodine alone (50 *μ*mol/L) or with simultaneous treatment with ryanodine and A23187 (50 *μ*mol/L and 1 *μ*mol/L, respectively. HDAC5‐labeled cells: control, *n* = 106; ryanodine, *n* = 122; ryanodine and A23187, *n* = 127). A representative image depicting a ryanodine + A23187‐treated, HDAC5‐labeled myocyte, clearly showing nuclear export of HDAC5. The results for Ca^2+^ signals are expressed as a background‐subtracted F/F_0_‐value and HDAC5 localization changes as a n/c‐ratio. All results are expressed as a mean ±SEM and the statistical significance calculated between control and each treatment using either the student's *t*‐test (HDAC5 translocation) or one‐way analysis of variance with Bonferroni's post hoc test (Ca^2+^ signals, * *P* < 0.05, ** *P* < 0.01, ***P < 0.001, scale bar 10 *μ*m).

Because SR Ca^2+^ release inhibition had such a strong effect on HDAC5 localization, the next aim was to determine if it is necessary for the translocation process, or if strong Ca^2+^ influx from any source can regulate [Ca^2+^]_nuc_ and trigger the HDAC5 redistribution. To study the effects of Ca^2+^ influx through the cell membrane on nuclear Ca^2+^ levels and HDAC localization, the membrane was permeabilized to Ca^2+^ using a Ca^2+^ ionophore, A23187 (1 *μ*mol/L), to enable strong Ca^2+^ influx. This caused a rapid increase in [Ca^2+^] throughout the cell, with the most pronounced increase observed within the nucleus (128% increase after 11 min, Fig. 4A), which also led to nuclear export of HDAC5 (Fig. 4B). Because in this experimental set‐up, CICR may have induced the increase in the nuclear Ca^2+^ levels, we next tested if A23187 is able to exhibit similar changes when SR Ca^2+^ release is blocked. A23187 could induce a strong nuclear Ca^2+^ influx even without the aid of SR Ca^2+^ release (Fig. 4C). These results suggest that [Ca^2+^]_nuc_ can be regulated by Ca^2+^ flux from either SR or through the cell membrane, as long as the increase of Ca^2+^ in the cytosol is sufficiently high.

Furthermore, A23187‐induced Ca^2+^ influx was able to rescue HDAC5 localization after SR Ca^2+^ release inhibition (Fig. 4D). HDAC5 localization is thereby dependent on the changing [Ca^2+^], not the source from where it is replenished. However, as the largest changes in [Ca^2+^] were observed within the nucleus, it is possible that the [Ca^2+^]_nuc_ changes may be specifically responsible for the changes in HDAC5 localization.

### Dynamics of HDAC5 Translocation

**Figure 5:**
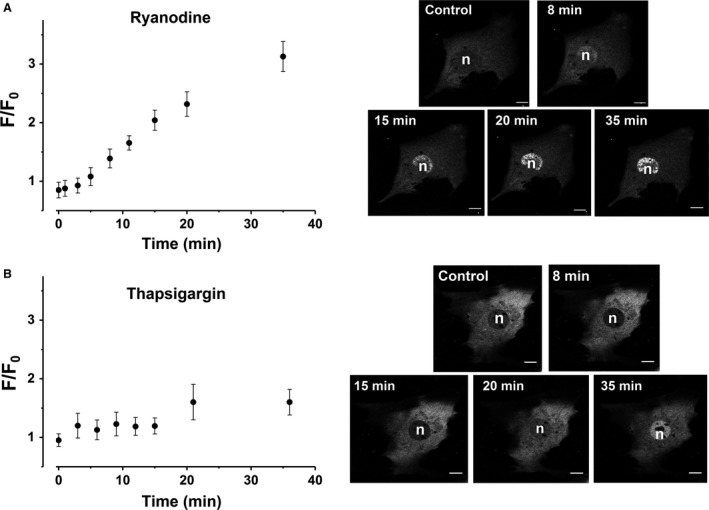
Dynamics of HDAC5 translocation. HDAC5‐GFP translocation with representative images at different time points in E9‐11 cardiomyocytes with (A) SR Ca^2+^ release inhibition (50 *μ*mol/L ryanodine, *n* = 9) and (B) with SERCA inhibition (10 *μ*mol/L thapsigargin, *n* = 23, scale bar 10 *μ*m). Electrical stimulation (0.5 Hz) was applied to the cells throughout the experiment. The mean, total change in the fluorescence intensity over the treatment period was 0.59 ± 0.16 arbitrary units (AU) with a rate of 0.016 ± 0.004 AU/min in thapsigargin treated, and 2.25 ± 0.24 AU and 0.062 ± 0.007 AU/min in ryanodine‐treated cells.

In HDAC5‐GFP transfected cells, HDAC5 was mainly found from the cytosol of spontaneously active cardiomyocytes. Inhibition of SR Ca^2+^ release (ryanodine, 50 *μ*mol/L, *n* = 9) and depletion of the SR Ca^2+^ stores (thapsigargin, 10 *μ*mol/L, *n* = 23) both caused a strong and relatively rapid nuclear accumulation of HDAC5‐GFP (Fig. 5A and B), despite the fact that the cells were electrically stimulated throughout the experiments. The average, total change in the fluorescence intensity over the treatment period was 0.59 ± 0.16 arbitrary units (AU), or 0.016 ± 0.004 AU/min in thapsigargin treated, and 2.25 ± 0.24 AU, 0.062 ± 0.007 AU/min in ryanodine‐treated cells. Such a substantial change in the rate of translocation could be explained by the different mode of action of the two drugs; ryanodine closes the SR RyR, hence almost immediately stopping spontaneous activity of the myocytes. Thapsigargin on the other hand is slower acting in stopping the spontaneous activity, as it blocks SR Ca^2+^ influx, hence spontaneous activity stops only when the SR Ca^2+^ stores are depleted.

Such dynamic shuffling between the nucleus and the cytosol gives HDAC5 the ability to function as an ON/OFF‐switch that can rapidly translocate upon changes in the amount and frequency of SR Ca^2+^ releases.

### SR Ca^2+^ release is essential for fetal cardiac‐specific gene expression

Inhibition of SR Ca^2+^ release significantly downregulated *Nkx2.5, MEF2c*, and *GATA4,* all of which are essential TFs in cardiac development and differentiation (Lints et al. 1993; Grepin et al. 1997; Kuo et al. 1997; Lin et al. 1997; Bi et al. 1999; Jamali et al. 2001). Also, changes in expression of the Ca^2+^ buffers Calsequestrin2 (*Casq*) and *Calr* were observed. *Casq* expression was downregulated, whereas *Calr* was upregulated upon SR Ca^2+^ release inhibition. Due to the developmental differences in *Calr* and *Casq* expression, where the latter takes over the role as the major Ca^2+^ buffering protein as the muscle matures (Mesaeli et al. 1999; Imanaka‐Yoshida et al. 1996) this differential change in their expression was expected.

**Figure 6:**
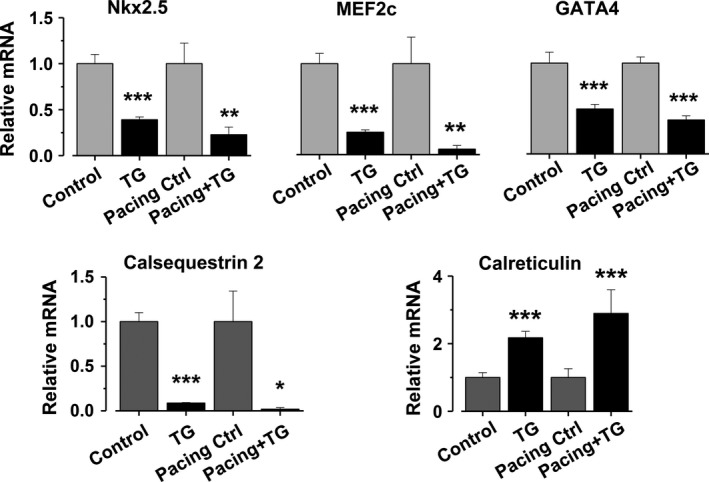
Expression of developmental phase‐dependent genes are regulated by [Ca^2+^]_nuc_. Cardiac‐specific gene expression in embryonic cardiomyocytes in spontaneously active (*n* = 6), thapsigargin‐treated (10 *μ*mol/L, *n* = 6), electrically stimulated (*n* = 4) samples and with simultaneous thapsigargin and electrical stimulation (*n* = 6). The cultures were treated overnight in order for the cells to have time to respond to the treatments at the gene expression level. Results are expressed as relative mRNA normalized to 18S, ±SEM. * *P* < 0.05, ** *P* < 0.01, *** *P* < 0.001

More importantly, electrical stimulation‐induced Ca^2+^ influx was not able to maintain the normal gene expression. The electrically stimulated cells showed similar down‐ and upregulation of the measured genes as the nonpaced controls (Fig. 6). These results further emphasize the essential role of the SR Ca^2+^ release as the main source of Ca^2+^ for regulating pathways that can up‐ or downregulate developmental phase‐dependent gene expression.

## Discussion

In this study, we show that in embryonic cardiomyocytes SR Ca^2+^ release is essential for inducing activity‐dependent Ca^2+^ signals into the nucleus. Without SR Ca^2+^ release nuclear Ca^2+^ is rapidly depleted, even with electrical stimulation‐induced opening of membrane Ca^2+^ channels (eg. the voltage‐activated Ca^2+^ channels, VACC). Significant changes in HDAC4 and HDAC5 intracellular distribution and cardiac‐specific gene expression were observed when SR Ca^2+^ release was inhibited.

Ca^2+^ signaling has an essential role in the development of the early cardiac tissue and the first asynchronous Ca^2+^ oscillations can be observed already at the early stages of cardiac crescent development. At this stage, no sarcomeric banding or myocyte contraction can be observed (Tyser et al. 2016). Such an order of appearance, with Ca^2+^ oscillations appearing prior to contractile elements, suggests that Ca^2+^ signaling at very early stages of development may have a role in guiding cardiomyocyte differentiation.

Nuclear Ca^2+^ signaling has been studied in various cell types, but the debate continues how it is maintained and regulated and if the nucleus is able to control its signaling alone, without input from the cytosol. The nuclear envelope has been shown to be a part of a single, continuous compartment with the SR that can function as a Ca^2+^ storage (Wu and Bers 2006). Furthermore, the cytosolic SR, perinuclear region, and even the nucleoplasmic reticulum have been shown to harbor both RyRs and IP_3_Rs (Ibarra et al. 2014; Guatimosim et al. 2008; Bers and Shannon 2013; Ljubojevic and Bers 2015). At the heart of the E9‐11 embryo, SR Ca^2+^ release is the major source of Ca^2+^ and the major mechanism for pacemaking in differentiating myocytes (Rapila et al. 2008; Karppinen et al. 2014; Korhonen et al. 2008; Sasse et al. 2007; Viatchenko‐Karpinski et al. 1999). Its perinuclear dominance (Korhonen et al. 2010; Rapila et al. 2008) also strongly suggests a role in nuclear Ca^2+^ signaling. However, whether its role is to stimulate Ca^2+^ release from the nuclear envelope, or simply to increase nuclear Ca^2+^ levels by increasing the cytosolic Ca^2+^ concentration that leads to diffusion to the nucleus, remains to be determined.

To accurately study differences in Ca^2+^ concentration between various intracellular localizations, calibration of the Ca^2+^‐binding dye to the localizations of interest is necessary. This is due to different Ca^2+^‐binding affinities of the different subcellular compartments, hence direct comparison of Ca^2+^ signals coming from different intracellular localizations without calibration of the dye is not possible. In this study, however, our interest was not to compare the changes between different localizations, but rather look at what happens to the relative fluorescence intensity of the Ca^2+^‐binding dye within the nucleus if SR Ca^2+^ release is inhibited.

Augmented perinuclear Ca^2+^ release through IP_3_R has been shown to specifically increase [Ca^2+^]_nuc_ and consequently alter localization of HDAC4 and HDAC5 in mature cardiomyocytes, thereby regulating cardiac‐specific gene expression (McKinsey 2007; Wu et al. 2006; Frey and Olson 2003; Zima et al. 2007; Zhang et al. 2002; Kockskamper et al. 2008; Peng et al. 2009). However, class IIa HDACs are of interest not only because of their important role in regulating gene expression in mature cardiomyocytes. Histone acetylation (H3K27) of the enhancer regions of genes involved in cardiomyocyte differentiation is essential in maintaining the open chromatin status (Wamstad et al. 2012). This enables transcription factor binding and expression of a pattern of cardiac genes during cardiac development. Hence, stimulation of HDAC5 nuclear import would have a strong effect on cardiomyocyte differentiation, as they could rapidly deacetylate these open chromatin regions, thereby repressing expression of genes essential for cardiomyocyte growth and differentiation.

In embryonic cardiomyocytes, Ca^2+^ signals that trigger contraction originate from the perinuclear SR and can thereby also serve as signals for nuclear‐specific Ca^2+^ signaling. Interestingly, the IP_3_R‐mediated Ca^2+^ release, which has been shown to increase nuclear Ca^2+^ levels in adult cardiomyocytes, has also been shown to maintain the whole‐cell Ca^2+^ transients in E9‐11 cardiomyocytes (Karppinen et al. 2014). However, because the perinuclear space is occupied with a large volume of SR and has an abundance of Ca^2+^ release units, SR Ca^2+^ release can either propagate into the nucleus or stimulate nuclear envelope Ca^2+^ release during each excitation. SR Ca^2+^ release was indeed shown to be the main regulator of nuclear Ca^2+^ levels in differentiating cardiomyocytes. With decreasing SR content (depletion with thapsigargin), we observed strong changes in Ca^2+^ distribution with the diminishing Ca^2+^ oscillations.

Previous studies have shown that SR Ca^2+^ release‐mediated changes in [Ca^2+^]_nuc_ can regulate expression of the cardiac‐specific TFs Nkx2.5, GATA4 and MEF2C (Kee and Kook 2011; Molkentin et al. 1998) which can be directly mediated by nuclear class IIa HDACs (Karamboulas et al. 2006; Kee and Kook 2011). Class IIa HDACs also regulate gene expression indirectly, by interacting with TFs, such as MEF2, thereby affecting expression of their target genes (Backs and Olson 2006; Mathiyalagan et al. 2014). In this current study, changing Ca^2+^ levels with subsequent HDAC translocation to nucleus was indeed associated with repression of these developmentally important TFs. A significant decrease in expression of Nkx2.5, GATA4, and MEF2C was observed even when the cells were electrically stimulated. This is in line with previous studies showing a vital role for HDAC in repressing the fetal cardiac genes (Wu et al. 2006). Furthermore, changes observed in expression of the two Ca^2+^ buffering proteins, CASQ2 and Calr, were quite interesting. Calr, which is found from the earlier embryonic tissues (Mesaeli et al. 1999), was found to be upregulated. However, CASQ2 expression was significantly decreased with SR Ca^2+^ release inhibition. This supports our hypothesis that a sufficiently high Ca^2+^ concentration is required for differentiation and maturation of the early muscle; increased Calr and decreased CASQ2 expression suggests the cells may be regressing toward a more immature phenotype.

Although HDACs are not the only regulators of cardiac‐specific gene expression, their role as a direct regulator of chromatin open/closed status is unmistakable (Wamstad et al. 2012). Other factors, such as nuclear factor of activated T‐cells (NFAT), can also be regulated by changing [Ca^2+^] and have a significant role in stimulation of cardiac‐specific gene expression (Arantes et al. 2012; Molkentin et al. 1998; Colella et al. 2008). However, no TFs can function unless the chromatin structure is open for them to bind. Hence, histone acetylation/deacetylation has a very special role in regulating expression of the early cardiac‐specific genes.

During the early developmental stages, strict temporal regulation of developmental‐specific gene expression is necessary for the tissue development to ensue in a correct manner. Therefore, a dynamic regulation of class IIa HDAC localization is to be expected, as the genes need to be repressed fast if, for example, activity of the cell changes. Our results do indeed show this, as uniform intracellular distribution of HDAC5 can be changed to mostly nuclear rapidly, due to the ability of differentiating myocytes to rapidly modify their [Ca^2+^] and Ca^2+^ distribution. A change in HDAC4 localization was also observed, but instead of a clear‐cut nucleus‐to‐cytosol translocation, accumulation of HDAC4 mostly into the nuclear periphery was observed. These results are quite interesting because previous studies have shown that HDAC4 and HDAC5 oligomerize and are co‐transported between the nucleus and the cytosol (Backs et al. 2008). Such different localizations of these two HDACs suggests they may have distinct roles during early development. Nuclear localization of HDAC4 suggests that it is actively involved in regulation of developmental phase‐specific cardiac genes, whereas the distribution pattern of HDAC5 implies it may have a smaller role during these early developmental stages. However, HDAC5 can be rapidly moved to the cytosol upon changes in Ca^2+^ homeostasis. Therefore, HDAC5 may function as a reserve that can rapidly move in to silence expression of its target genes if the activity, and with it the intracellular Ca^2+^ distribution, in embryonic cardiomyocytes changes. However, HDAC5 may also have a cytosolic role. A study on various cancer cell lines and an immortalized rat cardiomyocyte cell line (H9C2) showed that HDAC5 can facilitate stabilization and nuclear accumulation of the hypoxia‐inducible factor 1*α* (HIF‐1*α*). This action was accomplished by cytosolic deacetylation of the heat shock protein 70 (Hsp70) and subsequent stimulation of a pathway that led to nuclear accumulation of HIF‐1*α*. This is a very interesting finding because hypoxia has been found to stimulate multiple different developmental pathways in the developing embryo (Chen et al. 2015). Hence, the endogenous cytosolic localization of HDAC5 may have other significant functional roles in the developing embryos in addition to regulation of the chromatin open/closed status.

In the embryonic cardiomyocytes, SR Ca^2+^ release can initiate spontaneous cardiomyocyte contraction and create strong Ca^2+^ gradients across the cell (Korhonen et al. 2010; Louch et al. 2015). Any, even a relatively short‐term, change in cytosolic Ca^2+^ homeostasis can have a significant effect on [Ca^2+^]_nuc_, and thereby on various Ca^2+^‐activated pathways, some of which can control the subcellular localization of HDAC4 and HDAC5. Furthermore, SR Ca^2+^ release was shown to have a significant role in regulation of cardiac‐specific gene expression. These results provide a potential link between SR Ca^2+^ release and cardiac development, thereby also shedding light on the embryonic lethal knock‐out models that disrupt SR Ca^2+^ release.

## Conflict of Interest

None declared.
